# Endometrial Stromal Hyperplasia: An Underrecognized Condition

**DOI:** 10.1155/2013/204082

**Published:** 2013-04-04

**Authors:** Efthimios Sivridis, Gerasimos Koutsougeras, Alexandra Giatromanolaki

**Affiliations:** ^1^Department of Pathology, University General Hospital of Alexandroupolis, 68100 Alexandroupolis, Greece; ^2^Department of Obstetrics and Gynaecology, University General Hospital of Alexandroupolis, 68100 Alexandroupolis, Greece

## Abstract

Hyperplasia of the endometrial stroma is a poorly recognized lesion, lacking widespread recognition with most, if not all, such cases sequestrated in the literature as endometrial stromal nodules or low-grade endometrial stromal sarcomas. In this paper, we describe three examples of “endometrial stromal hyperplasia” which have a remarkable morphological similarity with the normally proliferating endometrial stroma and the endometrial stromal neoplasms, but which also possess subtle, but sufficient, differences to justify their taxonomic separation.

## 1. Introduction

The hyperplastic process in the endometrium is, for the most part, diffuse involves both glands and stroma (simple hyperplasia), less frequently, is focal or multifocal, and affects exclusively endometrial glands (complex hyperplasia and usually atypical hyperplasia) [1]. Endometrial hyperplasias consisting solely of stromal cells was only exceptionally reported in the literature [2], largely because these lesions are not recognized as a pathological entity distinct from the endometrial stromal nodule and the low-grade endometrial stromal sarcoma. We report here three such cases of stromal endometrial hyperplasia and reveal its subtle, but unique, features, for which this form of hyperplasia merits separate consideration. 

## 2. Case 1

A 71-year-old woman was referred for evaluation of a thick endometrium detected by routine ultrasound as part of her annual check-up. The patient had a past medical history of left colectomy for adenocarcinoma of the caecum 3 years ago, and polypectomy for a tubulovillous adenoma of the large intestine 2 years ago. She suffered from arterial hypertension for several years. There was no history of hormonal therapy. The patient had two normal deliveries and two induced abortions. Hysteroscopic biopsy produced a 0.5 cm^2^ fragment of tissue together with a small endometrial polyp 0.7 cm at maximum diameter.

**Figure 1:**
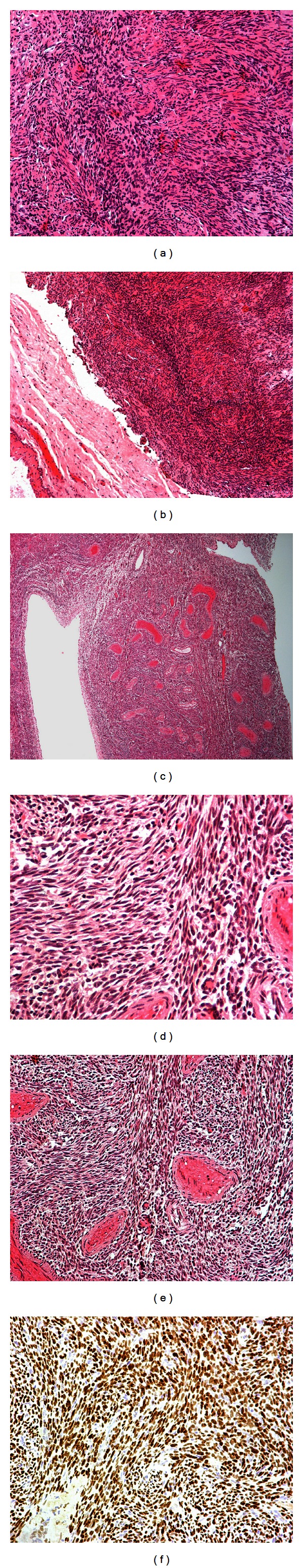
Endometrial stromal hyperplasia. (a) An endometrial fragment composed exclusively of small uniform spindle cells with scanty cytoplasm and ill-defined cell borders (H and E ×20). (b) The corresponding endometrial polyp showing a similar histological appearance (H and E ×10). (c) Endometrial stromal hyperplasia forming a small polyp composed of dense endometrial stroma with thick-walled vessels of the arteriolar type (H and E ×4). (d) The intraendometrial lesion of the same case at a higher magnification (H and E ×40). (e) Section from the polyp formed of spindle-shaped stromal cells and thick-walled vessels of the arteriolar type (H and E ×20). (f) Immunohistochemical expression of ER of the same case (×20).

On histological examination, the larger fragment was formed, almost entirely, of sheets of small uniform cells, with ovoid to spindle-shaped nuclei, having scanty cytoplasm and ill-defined cell borders (Figure 1(a)). There was a remarkable similarity with the stromal cells of a normal late proliferative type endometrium. The endometrial polyp contained a small area 0.2 cm in diameter, which was uniformly composed of dense endometrial stroma of similar type to that noted in the endometrial fragment (Figure 1(b)). Both specimens were free of cytological atypia, mitotic activity, or lymph-vascular space invasion. The biopsy was reported as “endometrial stromal lesion “stromal hyperplasia” endometrial stromal nodule.” The patient is free of any symptoms thereafter, and a subsequent ultrasound disclosed a normal thickness endometrium and myometrium. The patient is alive and well one year following treatment. In view of a normal ultrasound, the absence of any symptoms, and after review of the previously reported slides we regarded this case as endometrial stromal hyperplasia. 

## 3. Case 2

A 47-year-old woman was referred for gynaecological consultation by her family doctor because of a 30-month history of abnormal vaginal bleeding. She had two children, both delivered by Caesarean section. Physical examination revealed multiple uterine leiomyomas. Her prior medical history was left ovariectomy for mucinous cystadenoma 5 years ago. She was not receiving hormonal therapy. Total abdominal hysterectomy and right salpingo-oophorectomy were performed. 

The hysterectomy specimen contained multiple typical leiomyomas. There was widespread adenomyosis. The cervix was normal. The endometrium exposed a dense cellular polyp, 0.6 cm in greatest diameter, composed entirely of small spindle-shaped cells with little cytoplasm and ill-defined cell borders (Figure 1(c)). Thick-walled vessels of the arteriolar type were conspicuous throughout the lesion. A morphological similar lesion, with vessels of similar type, was seen in the endometrium over an area of 0.8 cm expanding into surrounding myometrium with a smooth pushing border (Figure 1(d)). There was no cytological atypia, mitotic activity, or lymph-vascular space invasion in either of the two lesions. The case was diagnosed as endometrial stromal hyperplasia. The right ovary showed a mucinous cystadenoma. The patient remains in good health for three years following removal of the uterus. 

## 4. Case 3

A 75-year-old woman presented with a one-week history of heavy abnormal vaginal bleeding. She had two normal deliveries. The patient was obese, hypertensive and had a 10 mm thick endometrium with two small polyps protruding into the endometrial cavity. She was currently taking thyroxin for hypothyroidism; she was not receiving hormonal therapy. Diagnostic and therapeutic curettage yielded four endometrial fragments composed entirely of small, regular stromal cells with ovoid to spindle-shaped nuclei and an ill-defined cytoplasm, reminiscent of normal late proliferative stroma. There were no cellular pleomorphism, mitotic activity, or invasion of lymph-vascular channels. The cells were arranged in sheets in which many vessels of the arteriolar cell type were present (Figure 1(e)). Of the two polyps received, one contained proliferative-type glands in a fibrotic stroma; the other was formed exclusively of stromal cells with features similar to those described in the endometrium. The biopsy was reported as “endometrial stromal lesion “stromal hyperplasia” endometrial stromal nodule.” A subsequent ultrasound was normal. The patient was alive and well at 14 months. On the basis of clinical and histological features, sonographic imaging, and follow-up information, this case was considered to be an endometrial stromal hyperplasia.

Immunohistochemistry was performed in all cases and revealed a diffuse positivity for ER (Figure 1(f)), PR, vimentin and bcl-2, and focal positivity for CD10. They were negative for *α*-smooth muscle actin, calretinin. 

## 5. Discussion 

There is now additional evidence that cellular proliferations in the endometrium may take the form of pure stromal hyperplasia—a rare, but specific, pattern of growth which is distinct from other forms of proliferative activity at this site. This is hardly surprising for a dynamic tissue, as the endometrium, having two main structural components, the endometrial glands and the specialized endometrial stroma, either of which, alone or in any combination, possesses the potentiality for intense proliferation and growth in response to appropriate hormonal stimuli. It has been recognized, in this respect, that prolonged unopposed oestrogenic stimulation of the endometrium may afford a pattern of hyperplastic growth which involves both endometrial components, that is, the glands and the stroma (simple hyperplasia), or it may, less frequently, induce a form of hyperplasia with proliferations restricted to endometrial glands (complex hyperplasia, atypical hyperplasia) [1]; analogous proliferations restricted to endometrial stroma have been identified, but this pattern of growth has been traditionally taken as being invariably neoplastic, either benign (endometrial stromal nodule) or malignant (low-grade endometrial stromal sarcoma, undifferentiated sarcoma)—a view which does not reflect the findings of the present report and apparently needs reconsideration. 

The belief that stromal cell proliferations in the endometrium are by definition neoplastic was first challenged by Stewart et al. [2] when they faced with a series of two endometrial biopsies and a subsequent hysterectomy specimen of a young woman with menorrhagia. The two biopsies showed a normal proliferative pattern endometrium admixed with small tissue fragments of dense endometrial stroma. The possibility of these being benign stromal tumours was considered in the biopsy material but excluded in the hysterectomy specimen where, for reasons to be discussed later, a diagnosis of focal endometrial stromal hyperplasia was made. This is the only fully documented case of endometrial stromal hyperplasia in the literature, and to this we have added the present three cases. A further three cases have been reported by Vanni et al. [3], while investigating translocations in endometrial polyps, but their report was brief and incomplete. 

The few examples of endometrial stromal hyperplasia recorded so far are only an approximation of its true incidence and for many cases have not been reported, while others remained unrecognized and recorded as endometrial stromal nodules or low-grade endometrial stromal sarcomas this is largely because the diagnostic label of stromal hyperplasia was not established. Nevertheless, these examples illustrate the point that “pure” stromal cell proliferations of the endometrium are, in itself, not necessarily neoplastic, but some may take the form of stromal hyperplasia. Yet, the recognition of this form of endometrial hyperplasia and its separation from the endometrial stromal tumours may present difficulties, particularly in curettage material, the hyperplastic stromal lesions are histologically very similar, almost identical, to those which are truly neoplastic, and there are only subtle morphological differences and molecular translocations that make the distinction possible. In fact, analysis of our cases showed several features to be of diagnostic value in this respect.

Thus, in accordance with Stewart et al. views [2], the endometrial stromal hyperplasias are entirely intraendometrial lesions, possibly extending into the myometrium with a pushing, rather than infiltrating, edge. The stromal cells forming the lesions, although very similar to those of the normal proliferative phase stroma, lack mitoses or show only occasional; they stain positively for CD10 and vimentin, express oestrogen (ER), and progesterone receptors (PR), but are negative for actin and desmin. The size of the lesions is smaller than 1 cm, and indeed, no case has been recorded in which the hyperplastic element was more than 0.8 cm diameter. Further, the stromal hyperplasias tend to form small polyps or to develop in a preexisting polyp which, in curettage specimens, may give an impression of multifocal involvement, as described by others [2]. Such endometrial polyps with a predominant pattern of stromal hyperplasia are characterized by *t(6; 14)(p21; q24)* translocation [3]. 

By contrast, neoplasms of the stromal nodule type are usually large, up to 20 cm in diameter, with an average size 5-6 cm diameter, although they can be as small as 0.8 cm [4]. These lesions may be intraendometrial or entirely intramural and are, with very few exceptions, solitary. Many are polypoid and distend the uterine cavity. The tumours are usually well circumscribed and expansile but, although often “projecting” up to 3 mm into the surrounding tissue, lack clear evidence of invasion. However, further extension into the myometrium, that is, beyond 3 mm, and/or clear evidence of vascular infiltration indicates that the tumour is an endometrial stroma sarcoma. The stromal nodules are compact, with cells that resemble those of the normally proliferating endometrial stroma, but may exhibit some nuclear atypia, and sparse mitotic activity: <3 mitoses per 10 high-power fields (HPF), but may sometimes reach 15 mitoses per 10 HPF. Common cytogenetic abnormality is a *t(7; 17)(p15q21)* that results in a *JAZF1-JJAZ1* gene fusion [5, 6]. 

The tumour cells of low-grade endometrial stromal sarcomas still resemble normal endometrial stromal cells, but may show mitotic activity (<3 mitoses to >10 mitoses per 10 HPF) and perhaps areas of nuclear pleomorphism. The distinctive feature of this tumour is, however, deep myometrial invasion, usually accompanied by invasion of vascular channels and rarely metastases. The tumours, in common with stromal nodules, show the fusion gene *JAZF1/JJAZ1* caused by the *t(7; 17)(p15; q21)* translocation [5, 6]. The undifferentiated endometrial sarcomas are frankly invasive tumours with haemorrhage, necrosis, and possibly metastases; they may show a greater degree of pleomorphism and mitotic activity (from ≥10 mitoses to ≥20 mitoses per 10 HPF), but lack distinctive molecular aberrations [5, 6]. 

The clinical features of patients with endometrial stromal hyperplasia are very much the same as those of women with stromal cell neoplasms, the commonest complaint being abnormal uterine bleeding. Similarly, the hyperplastic condition occurs over a wide age range, from before the 1940s to over 75 years of age, with a mean of 58 years. Following surgical removal of the uterus and ovaries, adenomyosis/endometriosis and uterine leiomyomas were commonly encountered, and, with the exception of Stewart's et al. case, there was a high incidence of coexistent endometrial polyps. These pathological features are in no way specific or characteristic for this particular form of hyperplasia, but it seems to imply that an oestrogenic milieu prevails. 

It is apparent from this discussion that pure stromal cell proliferations in the endometrium are not necessarily neoplastic, as the few examples described appear to be genuine hyperplastic in nature. It is suggested that if a stromal lesion is of small size, intraendometrial in localization, and multifocal in distribution, and if it has a tendency to form polyps or arise in a preexisting polyp and lacks vascular invasion, then it is justifiable to be considered as endometrial stromal hyperplasia. A specific molecular genetic test that might support the diagnosis in problematic cases is *t(6; 14)(p21; q24)*. Despite the short followed up time, it is believed that any hyperplastic lesion lacking cytological atypia and, for that matter, endometrial stromal hyperplasias have probably a favourable outlook and, therefore, merit taxonomic separation.
